# Supplementary material for: Level of dietary adherence and determinants among type 2 diabetes population in Ethiopian: A systemic review with meta-analysis
Source: PLoS One. 2022 Oct 10;17(10):e0271378. doi: 10.1371/journal.pone.0271378 (PMC9550051; doi:10.1371/journal.pone.0271378)
Supplement: S1 Table — (DOCX) [file pone.0271378.s002.docx]

**Supporting information 1**: Search strategy applied to PubMed database in the current review

| **Search #** | **Query** | **Items found** |
| --- | --- | --- |
| #1 | **dietary adherence OR recommended dietary practice OR therapy adherence OR treatment adherence OR medication intake adherence OR medication compliance OR patient compliance [MeSH Terms]** | 254,121 |
| #2 | **Diabetes mellitus OR type 2 diabetes OR diabetes [MeSH Terms]** | 447,096 |
| #3 | **Patients OR clients [all field]** | 7,402,997 |
| #4 | **Factors OR determinants OR influences OR risk factors OR predictors [MeSH Terms]** | 860,132 |
| #5 | **Ethiopia** | 25,115 |
| #6 | **#1 AND #2 AND #3 AND #4 AND #5** | 48 |
