# Supplementary material for: Level of dietary adherence and determinants among type 2 diabetes population in Ethiopian: A systemic review with meta-analysis
Source: PLoS One. 2022 Oct 10;17(10):e0271378. doi: 10.1371/journal.pone.0271378 (PMC9550051; doi:10.1371/journal.pone.0271378)
Supplement: S2 Table — (DOCX) [file pone.0271378.s003.docx]

| **Supporting information 2**. Scoring of the quality of articles by authors using the Newcastle-Ottawa Quality Assessment tool. | | | | | | | | | | |
| --- | --- | --- | --- | --- | --- | --- | --- | --- | --- | --- |
| Study | Quality assessors | Selection | | | | Comparability | | Outcome | | Total score |
|  |  | Representativeness of sample (*, *) | Sample size (*) | Non-respondents (*) | Ascertainments (**) | Study control for most important factors (*) | Study control any additional factors (*) | Assessment of the outcome (*) | Statistical test (*) |  |
| Tibebu A,et al | BM | 1 | 1 | 1 | 1 | 0 | 1 | 1 | 0 | 6 |
|  | TWA | 1 | 0 | 1 | 1 | 1 | 0 | 1 | 1 |  |
|  | AY | 1 | 1 | 0 | 1 | 1 | 1 | 0 | 1 |  |
|  | HG | 1 | 0 | 1 | 1 | 0 | 1 | 1 | 1 |  |
| Nadewu AN, et al | BM | 1 | 0 | 0 | 2 | 1 | 1 | 0 | 1 | 6 |
|  | TWA | 1 | 1 | 1 | 0 | 0 | 1 | 1 | 1 |  |
|  | AY | 1 | 1 | 1 | 1 | 1 | 1 | 0 | 1 |  |
|  | HG | 1 | 0 | 1 | 1 | 0 | 1 | 1 | 0 |  |
| Tesema S, et al | TE | 1 | 1 | 1 | 0 | 1 | 0 | 1 | 1 | 6 |
|  | TWA | 1 | 0 | 1 | 1 | 0 | 1 | 1 | 0 |  |
|  | YA | 1 | 1 | 1 | 1 | 1 | 1 | 1 | 1 |  |
|  | HG | 0 | 0 | 1 | 1 | 0 | 1 | 1 | 1 |  |
| Buda ES, et al | MB | 2 | 1 | 0 | 1 | 1 | 1 | 1 | 1 | 8 |
|  | TWA | 2 | 1 | 1 | 1 | 1 | 0 | 1 | 1 |  |
|  | AY | 1 | 1 | 0 | 1 | 1 | 1 | 1 | 1 |  |
|  | HG | 2 | 1 | 1 | 1 | 1 | 1 | 1 | 1 |  |
| Harei HA, et al | MB | 1 | 0 | 1 | 0 | 1 | 1 | 0 | 1 | 7 |
|  | TWA | 1 | 1 | 1 | 1 | 1 | 1 | 1 | 1 |  |
|  | AG | 1 | 1 | 1 | 1 | 1 | 1 | 1 | 1 |  |
|  | HG | 1 | 1 | 0 | 1 | 1 | 1 | 1 | 1 |  |
| Labata BG, et al | MB | 1 | 0 | 0 | 1 | 1 | 0 | 1 | 1 | 6 |
|  | TWA | 1 | 1 | 1 | 1 | 0 | 1 | 0 | 1 |  |
|  | AG | 0 | 1 | 1 | 1 | 1 | 1 | 1 | 1 |  |
|  | HG | 1 | 0 | 1 | 1 | 0 | 1 | 1 | 1 |  |
| WorkuKassahun C, et al | MB | 1 | 1 | 0 | 1 | 1 | 1 | 1 | 1 | 7 |
|  | TWA | 1 | 0 | 1 | 1 | 0 | 0 | 1 | 1 |  |
|  | AY | 1 | 1 | 0 | 1 | 1 | 1 | 1 | 1 |  |
|  | HG | 2 | 1 | 1 | 1 | 1 | 1 | 1 | 1 |  |
| Angelo AT, et al | MB | 1 | 1 | 1 | 1 | 1 | 1 | 1 | 1 | 8 |
|  | TWA | 2 | 1 | 1 | 1 | 0 | 1 | 1 | 1 |  |
|  | AY | 1 | 1 | 0 | 1 | 1 | 1 | 1 | 1 |  |
|  | HG | 2 | 1 | 1 | 1 | 1 | 1 | 1 | 1 |  |
| Fetensa G, et al | MB | 1 | 1 | 1 | 1 | 1 | 1 | 1 | 1 | 6 |
|  | TWA | 0 | 1 | 1 | 1 | 0 | 0 | 0 | 1 |  |
|  | AY | 1 | 0 | 0 | 0 | 1 | 1 | 1 | 0 |  |
|  | HG | 1 | 1 | 1 | 1 | 1 | 1 | 1 | 1 |  |
| Gebremichael G, et al | MB | 2 | 1 | 0 | 1 | 1 | 1 | 1 | 1 | 8 |
|  | TWA | 2 | 1 | 1 | 1 | 1 | 1 | 1 | 1 |  |
|  | AY | 1 | 1 | 0 | 0 | 1 | 1 | 1 | 1 |  |
|  | HG | 2 | 1 | 1 | 1 | 1 | 1 | 1 | 1 |  |
| Niriayo YL, et al | MB | 2 | 1 | 1 | 1 | 1 | 1 | 1 | 1 | 8 |
|  | TWA | 1 | 0 | 1 | 1 | 1 | 1 | 1 | 1 |  |
|  | AY | 2 | 1 | 1 | 1 | 1 | 1 | 1 | 1 |  |
|  | HG | 1 | 1 | 1 | 1 | 1 | 1 | 1 | 1 |  |
| Seid A,et al | MB | 2 | 1 | 1 | 1 | 1 | 1 | 1 | 1 | 8 |
|  | TWA | 2 | 1 | 1 | 1 | 0 | 1 | 1 | 1 |  |
|  | AY | 1 | 1 | 0 | 1 | 1 | 1 | 1 | 1 |  |
|  | HG | 1 | 1 | 1 | 1 | 1 | 1 | 1 | 1 |  |
| Ademe S, et al | MB | 1 | 1 | 1 | 1 | 1 | 1 | 1 | 1 | 6 |
|  | TWA | 1 | 0 | 1 | 1 | 0 | 0 | 0 | 1 |  |
|  | AY | 1 | 1 | 0 | 0 | 1 | 0 | 1 | 0 |  |
|  | HG | 1 | 1 | 1 | 1 | 1 | 1 | 1 | 1 |  |
| Seindew M, et al | MB | 1 | 1 | 1 | 1 | 1 | 1 | 1 | 1 | 6 |
|  | TWA | 1 | 0 | 1 | 0 | 0 | 1 | 0 | 1 |  |
|  | AY | 1 | 1 | 0 | 1 | 1 | 1 | 1 | 0 |  |
|  | HG | 1 | 0 | 1 | 1 | 1 | 0 | 1 | 1 |  |
| Gebre NT,et al | MB | 1 | 1 | 1 | 1 | 1 | 1 | 1 | 1 | 6 |
|  | TWA | 0 | 1 | 1 | 1 | 0 | 0 | 0 | 1 |  |
|  | AY | 1 | 0 | 0 | 0 | 1 | 1 | 1 | 0 |  |
|  | HG | 1 | 1 | 1 | 1 | 1 | 1 | 1 | 1 |  |
| Feleke M, et al | MB | 1 | 1 | 0 | 1 | 1 | 1 | 1 | 1 | 8 |
|  | TWA | 1 | 1 | 1 | 1 | 1 | 0 | 1 | 1 |  |
|  | AY | 2 | 1 | 1 | 1 | 1 | 1 | 1 | 1 |  |
|  | HG | 2 | 1 | 1 | 1 | 1 | 1 | 1 | 1 |  |
| Takele N, et al | MB | 1 | 1 | 1 | 1 | 1 | 1 | 1 | 1 | 7 |
|  | TWA | 1 | 0 | 1 | 1 | 0 | 1 | 1 | 1 |  |
|  | AY | 1 | 1 | 0 | 1 | 1 | 0 | 1 | 1 |  |
|  | HG | 1 | 1 | 1 | 1 | 1 | 1 | 1 | 1 |  |
