# Supplementary material for: Level of dietary adherence and determinants among type 2 diabetes population in Ethiopian: A systemic review with meta-analysis
Source: PLoS One. 2022 Oct 10;17(10):e0271378. doi: 10.1371/journal.pone.0271378 (PMC9550051; doi:10.1371/journal.pone.0271378)
Supplement: S3 Table — (DOCX) [file pone.0271378.s004.docx]

**Supporting information** 3: Risk of Bias assessment Tool of Eligible Articles by using the Hoy 2012 tool

| Study | Representation | Sampling | Random selection | Non response bias | Data collection | Case Definition | Reliability and validity of study tool | Method of data collection | Prevalence period | Numerator and denominator | Summary Assessment |
| --- | --- | --- | --- | --- | --- | --- | --- | --- | --- | --- | --- |
| Tibebu A,et al | Low risk | Low risk | Low risk | Low risk | Low risk | Low risk | High risk | High risk | low risk | Low risk | Low risk |
| Nadewu AN, et al | Low risk | Low risk | Low risk | Low risk | Low risk | Low risk | High risk | High risk | low risk | Low risk | Low risk |
| Tesema S, et al | Low risk | Low risk | Low risk | Low risk | Low risk | High risk | Low risk | Low risk | Low risk | Low risk | Low risk |
| Buda ES, et al | Low risk | Low risk | Low risk | Low risk | Low risk | Low risk | Low risk | Low risk | Low risk | Low risk | Low risk |
| Harei HA, et al | Low risk | Low risk | High risk | Low risk | High risk | High risk | High risk | Low risk | Low risk | Low risk | Medium risk |
| Labata BG, et al | Low risk | Low risk | High risk | Low risk | Low risk | Low risk | Low risk | Low risk | Low risk | Low risk | Low risk |
| WorkuKassahunC , et al | High risk | Low risk | High risk | Low risk | High risk | Low risk | High risk | Low risk | Low risk | Low risk | Medium risk |
| Angelo AT, et al | High risk | Low risk | Low risk | Low risk | Low risk | Low risk | Low risk | Low risk | Low risk | Low risk | Low risk |
| Fetensa G, et al | High risk | Low risk | Low risk | Low risk | Low risk | Low risk | Low risk | Low risk | Low risk | Low risk | Low risk |
| Gebremichael G, et al | High risk | High risk | Low risk | Low risk | Low risk | Low risk | Low risk | Low risk | Low risk | Low risk | Low risk |
| Niriayo YL, et al | Low risk | Low risk | Low risk | Low risk | Low risk | Low risk | Low risk | Low risk | Low risk | Low risk | Low risk |
| Seid A,et al | High risk | Low risk | Low risk | Low risk | Low risk | High risk | Low risk | Low risk | Low risk | Low risk | Low risk |
| Ademe S, et al | High risk | Low risk | Low risk | High risk | High risk | High risk | High risk | Low risk | High risk | Low risk | High risk |
| Sindew M, et al | High risk | Low risk | High risk | High risk | Low risk | Low risk | Low risk | Low risk | Low risk | Low risk | Low risk |
| Gebre NT,et al | Low risk | Low risk | Low risk | Low risk | Low risk | Low risk | Low risk | Low risk | Low risk | Low risk | Low risk |
| Feleke M, et al | Low risk | Low risk | Low risk | Low risk | Low risk | Low risk | Low risk | Low risk | Low risk | Low risk | Low risk |
| Takele N, et al | High risk | High risk | High risk | Low risk | Low risk | High risk | Low risk | Low risk | Low risk | Low risk | Low risk |
| **Risk of bias assessment tool: Yes (low risk); No (high risk)** | | | | | | | | | | |  |
| 1. Representation: Was the study population a close representation of the national population? | | | | | | | | | | |  |
| 2. Sampling: Was the sampling frame a true or close representation of the target population? | | | | | | | | | | |  |
| 3. Random selection: Was some form of random selection used to select the sample OR was a census undertaken? | | | | | | | | | | |  |
| 4. Non-response bias: Was the likelihood of non-response bias minimal? | | | | | | | | | | |  |
| 5. Data collection: Were data collected directly from the subjects? | | | | | | | | | | |  |
| 6. Case definition: Was an acceptable case definition used in the study? | | | | | | | | | | |  |
| 7. Reliability and validity of study tool: Was the study instrument that measured the parameter of interest show to have reliability and validity? | | | | | | | | | | |  |
| 8. Data collection: Was the same mode of data collection used for all subjects? | | | | | | | | | | |  |
| 9. Prevalence period: Was the length of the prevalence period for the parameter of interest appropriate? | | | | | | | | | | |  |
| 10. Numerators and denominators: Were the numerator(s) and denominator(s) for the parameter of interest appropriate? | | | | | | | | | | |  |
| The overall risk of bias scored based on the number of high risks of bias per study: low risk (≤2), moderate risk (3–4), and high risk (≥5). | | | | | | | | | | |  |
