# Supplementary material for: Level of dietary adherence and determinants among type 2 diabetes population in Ethiopian: A systemic review with meta-analysis
Source: PLoS One. 2022 Oct 10;17(10):e0271378. doi: 10.1371/journal.pone.0271378 (PMC9550051; doi:10.1371/journal.pone.0271378)
Supplement: Attachment — Submitted filename: review_comment_ethiopia-diabates_feb2022.pdf [file pone.0271378.s005.pdf]

Page	Line	Comments
2	12	Were the “independent reviewers” enough experienced to review? They should be well-experienced reviewers, so it would be better to mention their level.
4	16	Change “Is” to “was”. Please proofread the manuscript again to revise minor mistakes.
5	5	Remove one “with”. There are two “with”.
5	6	Why the participants aged 30 and above were considered an inclusion criteria? This is not consistent with the analyzed study target generation.
6	4-5	“find articles by reviewing the 5 reference lists of already identified researches.” This sentence is not easy to understand. Please rephrase.
6	18	How did you assure the third independent review’ quality?
6	22	How did you assure the “Discrepancies between reviewers resolved by discussion”? How was the discussion conducted?
7	17	Please rephrase “the risk of bias of included studies”. It is difficult to follow.
8	1-2	“For 2 the least risk of bias classification, discrepancies between the reviewers resolved via consensus” How did you assure the validity? Did you use any references? The words of “resolved via concensus” is not clear how the process was conducted systematically and analytically.
8	14	“study setting (hospitals)” is this type of hospital? Please rephrase to make it clear.
9	1	Why was P-value set at 0.1, not 0.05? P-value of 0.05 has more significant than that of 0.1 statistically.
9	4	“Eff ect” and “aff ected” It looks there are redundant space between f and e.
10	24	“participants (aged 15-85 years) “ In the eligibility criteria section, it was mentioned that "Participants aged 30 years old and above were also considered an inclusion criterion." Why the age criteria is inconsistent with the eligibility criteria?
11	4	Why did not you exclude two studies? How did you assure the quality of the results of the non-peer reviewed studies?
11	5	The response rate was quite high. Any bias? Did all studies take appropriate ethical consideration measure?
12	11	“study setting (hospital)” Same comment to page 8 line 14.
14	2	Mistypo “estimatio”
15	4-5	Please rephrase “Egger’s test of the intercept 5 was 0.291 (95% CI: -0.085, 0.667) p > 0.05 (0.121) as judged by Egger’s test”. It is not easy to follow.
21	5-6	What is the evidence of “Dietary adherence emphasizes the importance of minimizing macro-vascular and micro6 vascular complications in people with diabetes.”
21	9	What is these “interventions”? There are various interventions for DM control.
21	19	The knowledge of healthy diet behaviour would be different from the knowledge gained at primary/junior high schools. Why we can say this relation?
22	1	It should be that low-income participants are prone to follow unhealthy diet. It is not consistent with the findings.
22	1	Why "emotional distress" is raised here suddenly? What is the meaning of "emotional distress" here?
22	5-6	“the cost of healthy diets increased with increasing 6 income quintiles” This is not clear. The expenditure of healthy diets?
22	7	What is the level of knowledge compared to a low level of it?
22	14	What is the difference of simple sugar and "sugar"? If there is no difference, it would be better to rephrase "sugar".

22	17	Why "nutrition knowledge" is started to discuss here. The main theme should be summarized at the last paragraph. “nutrition knowledge” should not be fully equal to dietary adherence and healthy diet behavior for type 2 DM.
23	1	What is the several strengths? I can identify only one strength of the sample size. Please mention clearly several strengths, if you want to say them here.
23	4-5	What is the result of the trial? Just "tried" cannot be the stength of this study.
23	7	Please rephrase “all primary studies were cross-sectional that is limited in this study”. It is not easy to follow.
23	7-9	What is the results of your trial? As the result, what is the limitation? Please rephrase to make clear the limitation. “Besides, we 8 tried extensive and diverse search strategies to find all possible available literature, some grey 9 literature, such as conference proceedings, remained difficult to find.”
23	13-14	What kind of “effective strategies” could be suggested from this study? Please describe.
23	16-17	“Future research should focus on development” What for is “development”?
23	17-18	“Testing recommends food availability, affordability, and cultural acceptability in the 18 context of Ethiopians.” Please rephrase. What is “testing”?
23	23	What is the evidence of “based on what they see, hear, feel, and perceive the healthy diet”?
24	2-3	Please rephrase “Ensure more focusing on eating a healthy diet is as preventive and curative measures for 3 diabetes in health education programs as well in medical curricula.” to make the meaning clear.
24	23-	The style of Reference does not follow the guideline. Please recheck the style.
